# An Enhanced Particle Swarm Optimized RBF Model for Precise Fish Population Estimation in Cage Farming

**DOI:** 10.3390/ani16132057

**Published:** 2026-07-03

**Authors:** Gang Yang, Xuelei Wang, Junping Wang, Weiliang Shen, Hongsheng Yang, Qingfei Li, Chenggang Lin

**Affiliations:** 1 CAS Key Laboratory of Marine Ecology and Environmental Sciences, Institute of Oceanology, Chinese Academy of Sciences, Qingdao 266071, China; yg958546257@gmail.com (G.Y.); hshyang@qdio.ac.cn (H.Y.); 2 Ningbo Academy of Oceanology and Fisheries, Ningbo 315012, China; xlwang126@163.com (X.W.); sweleon@163.com (W.S.); 3 School of Marine Science and Engineering, Qingdao Agricultural University, Qingdao 266109, China; 13573939865@163.com

**Keywords:** feed intake, bio-environmental data, bioenergetics, fish population estimation, machine learning

## Abstract

In cage aquaculture, precise estimation of fish biomass is critically important for determining appropriate feeding strategies and evaluating production capacity. Currently, prevailing fish counting approaches heavily rely on acoustic or optical technologies. However, the accuracy and reliability of the obtained data are largely compromised by factors such as fish occlusion and water turbidity in practical cage farming environments. To address this limitation, this study proposed a novel method for estimating fish population size deduced from dynamic feeding information, based on the model integrated environmental and biological factors, feed intake and biomass. A 10-week feeding experiment was carried out to collect multidimensional data including feed intake, growth parameters, and environmental variables to construct a dataset correlating feeding amount with primary influential factors. Herein a bioenergetics-informed radial basis function neural network, optimized via particle swarm optimization (BE-PSO-RBF), was developed based on those empirical data. Model validation using 47 independent test samples showed that the hybrid model achieved a mean absolute error (MAE) of 26.82, a root mean square error (RMSE) of 35.62, and a mean absolute percentage error (MAPE) of 4.14%, confirming its robust generalization performance. These findings suggest that feed-intake-based population estimation may provide a feasible complementary approach for fish population assessment under cage farming conditions similar to those investigated in this study.

## Simple Summary

Estimating the number of fish in sea cages is essential for efficient feeding and sustainable aquaculture management. Most existing methods rely on underwater cameras or acoustic devices, but their performance can be affected by poor water visibility, fish crowding, and high equipment costs. In this study, we developed a new method that estimates fish population size using feeding information together with fish growth and environmental conditions. A 10-week feeding experiment with large yellow croaker was conducted to collect biological, feeding, and environmental data for model development and evaluation. The proposed method accurately estimated fish population size without requiring continuous underwater imaging or acoustic monitoring. Because feeding information is routinely recorded in commercial aquaculture, this approach could provide farmers with a practical and cost-effective tool for monitoring fish populations and supporting daily feeding management. Although further validation under different farming conditions and for additional fish species is still needed, this study demonstrates that feeding-based estimation is a promising complementary approach for precision cage aquaculture.

## 1. Introduction

Precision management within deep-water cage systems has become the fundamental prerequisite for the viability and advancement of the contemporary aquaculture industry [1]. For any type of aquaculture, the robust estimation of population size is a critical determinant for optimizing feeding regimes and maximizing production efficiency [1]. Traditional manual counting methods are not only labor-intensive and time-consuming, but repeated handling can also cause significant stress responses and physical damage to fish, thereby limiting their applicability to large-scale and technology-oriented aquaculture operations [2,3]. With rapid advances in artificial intelligence and data acquisition technologies, traditional aquaculture is increasingly transitioning toward intelligent and automated production systems [4]. Therefore, the development of intelligent, non-invasive population estimation frameworks has emerged as a vital trajectory for advancing modern cage aquaculture practices [5].

With the development of deep learning, fish population estimation in cage aquaculture is increasingly transitioning from manual counting to automated, data-driven approaches [6]. To date, most fish counting methods have been developed based on optical or acoustic sensing technologies [7]. Nishikawa et al. (2025) proposed a counting method based on fishfinder echo images combined with a convolutional neural network (CNN), reporting an estimation error ranging from 0.86% to 6.89% [8]. Helminen et al. (2021) employed an adaptive resolution imaging sonar (ARIS) system to achieve automatic fish counting in river environments [9]. Xing et al. (2024) further developed a sonar detection system by integrating an improved YOLOv8 framework with the BoT-SORT tracking algorithm, which increased the recall rate by 3.8% [10]. Liu et al. (2021) proposed a fish segmentation framework designed for deep-sea cage aquaculture, using multi-scale Gaussian modeling and direction-weighted convolution kernels to enable accurate counting of high-density fish populations [11]. In recirculating aquaculture systems (RAS), several studies have also explored computer vision-based approaches. For example, Zhu et al. (2025) combined the VGG19 neural network with an adaptive attention mechanism, which effectively mitigated the influence of fish occlusion and body size variation on counting accuracy, achieving an accuracy of 95.81% [12]. Du et al. (2023) proposed a fish counting model that integrates multi-column dilated convolution, an attention mechanism and the Swin Transformer architecture, and reported an accuracy of 97.57% under complex and high-density conditions [13]. In another study, Zhu et al. (2024) developed a density estimation network named FCFormer, which reduces the impact of fish occlusion through improved feature representation and achieves a counting accuracy of 97.06% [14].

However, despite these algorithmic and machinery advancements, existing methodologies remain primarily reliant on isolated sensing modalities, rendering them susceptible to environmental interference in deep-water cages [13]. For example, optical monitoring approaches often experience a significant decline in detection performance when water turbidity increases or when the contrast between fish and the background is low [14]. In addition, it is difficult for such systems to achieve sufficient coverage in large-scale farming areas. While acoustic telemetry partially mitigates visual limitations, high capital costs and signal interference from non-target biomass—such as macroalgae—hinder its scalability and reliability in practical open-water environments [15]. Given these limitations, it is necessary to explore alternative approaches for estimating fish population size that are less affected by water conditions and fish density, while maintaining greater stability and adaptability in practical aquaculture environments.

In cage aquaculture, teleost appetitive behavior is a multi-factorial process modulated by species-specific traits, ontogenetic development, and physiochemical environmental forcing [16]. Consequently, the cumulative feed intake within a production unit is closely associated with total population biomass, offering a robust biological proxy for non-invasive, indirect stock assessment [17]. Given that, leveraging feed intake dynamics as a deterministic biological proxy offers a robust alternative for indirect population estimation, bypassing the environmental limitations of unimodal sensing [18]. Neural networks have advantages in dealing with complex nonlinear relationships and can learn potential patterns from data under multi-variable conditions, providing a feasible approach for fish population estimation [19]. Based on this, the present study collected multidimensional biological and environmental data using intelligent sensing devices and applied a neural network model to describe the relationship between feeding behavior and fish population size.

## 2. Materials and Methods

### 2.1. Experimental Fish

The experimental animals used in the present study were large yellow croaker (*Larimichthys crocea*), obtained from an aquaculture farm in Ningbo, Zhejiang Province, China. A total of four sea cages were established and designated as A, B, C, and D. Cages A–C were used for the construction of the fish stock size prediction (FSSP) dataset, whereas cage D was used to establish the fish feeding influencing factors (FFIF) dataset. Detailed information regarding the experimental cages is provided in Table 1.

### 2.2. Rearing Method

The experiment was conducted in high-density polyethylene (HDPE) raft-type sea cages at a coastal aquaculture farm in Ningbo, Zhejiang Province, China. A total of four experimental cages (A, B, C, and D) were included in the study, all of which were identical in size and measured 9.0 m × 4.5 m × 3.6 m (length × width × depth).

The feeding trial was carried out over a period of 10 weeks. Fish were fed twice daily at 05:00 and 17:00, and each feeding session was divided into two consecutive rounds. During each feeding event, floating pellet feed was delivered using a fixed-point feeding method. Throughout the experimental period, the mean water temperature, dissolved oxygen concentration, and pH in the farming area were 20.18 ± 3.02 °C, 8.50 ± 0.48 mg/L, and 8.64 ± 0.04, respectively (mean ± SD).

### 2.3. Data Collection and Preprocessing

#### 2.3.1. Fish Population Size and Stocking Density Collection

Throughout the experimental period, 80, 20, and 20 large yellow croakers were randomly removed from cages A, B, and C, respectively, at 22:00 every two days and transferred to non-experimental cages. No fish removal was conducted in cage D. Mortality was inspected and recorded daily throughout the experimental period, and dead fish were removed immediately after observation. The ground-truth fish population size used for model training and validation was updated based on the known initial stocking number, the recorded number of fish removed during each sampling event, and the daily mortality records. Accordingly, the fish population size and stocking density of each cage for the following day were calculated as follows:

(1) $$N_{t+1}\;=\;N_{t}-R_{t}-M_{t}$$

(2) $$D_{t+1}=\frac{N_{t+1}}{V_{c}}$$

where $N_{t}$ is the actual fish population on day t, $R_{t}$ is the number of fish removed on day t, $M_{t}$ is the number of mortalities recorded on day t, and $N_{t+1}$ is the actual fish population on the following day, $D_{t+1}$ is the stocking density on the following day, and $V_{c}$ is the volume of the experimental cage.

#### 2.3.2. Feed Intake Determination

A standardized satiation-based feeding protocol was implemented throughout the experiment. Floating pellet feed was used, and each cage was equipped with a feed-retaining enclosure to reduce pellet drift during feeding. A two-stage feeding regime was established and applied during the daily operations and data collection. During the initial feeding phase, administration ceased upon a discernible decline in appetitive intensity, signaled by the emergence of residual pellets on the water surface. Following a 20-min refractory period, a secondary feeding phase was launched and maintained until terminal satiation, defined by a fixed residual feed threshold of approximately 10–20 g. If no vigorous feeding behavior was observed within a 5-min latency window, the feeding session was terminated. The cumulative amount of feed dispensed across both phases was then recorded as the total feed intake for the single day.

#### 2.3.3. Age and Body Weight Collection

The day of hatching was designated as 1 day post-hatch (1 DPH), and chronological age was determined accordingly with reference to the feeding schedule. In view of the relatively limited growth heterogeneity under stable feeding conditions, body weight was measured on a weekly basis throughout the experimental period. During each sampling event, 50 fish per cage were randomly obtained by gently crowding the stock to one side of the cage with the net. The collected fish were anesthetized in seawater supplemented with 130 ppm MS-222 prior to weighing. Individual body weight was then recorded for each fish, and the average body weight of each cage was derived as the arithmetic mean of the sampled individuals. Upon completion of the measurements, the fish were transferred to clean, aerated seawater for recovery and subsequently returned to their original cages.

#### 2.3.4. Water Quality Parameter Collection

An FB_300_ZKHYS buoy system was installed in the experimental cage to enable the continuous acquisition of water quality data throughout the experimental period. Environmental parameters, including water temperature, dissolved oxygen, conductivity, pH, salinity, and chlorophyll concentration, were recorded automatically at 20-min intervals. After the initial feeding phase each day, the monitored data were retrieved via a PC-based monitoring platform and subsequently incorporated into the downstream analyses.

#### 2.3.5. Hydrodynamics and Light Intensity Collection

Water flow velocity within the cage was determined using an LS300-A handheld current meter (Nanjing Ouka Instrument Co., Ltd., Nanjing, China). Light intensity was quantified simultaneously with a DL333205 digital illuminance meter (Deli Group Co., Ltd., Ningbo, China). Both measurements were carried out during each feeding session to maintain procedural consistency in the collected data.

#### 2.3.6. Meteorological and Tidal Information Collection

Meteorological and tidal data relevant to the farming site were retrieved from the Global Tidal Information System (https://global-tide.nmdis.org.cn (accessed on 30 September 2023)) and the China Marine Forecasting Platform (https://www.oceanguide.org.cn (accessed on 30 September 2023)). The collected variables comprised wave height, tidal level, and the trend of tidal fluctuation corresponding to each feeding session, and they were subsequently incorporated into the environmental dataset for further analysis.

#### 2.3.7. Data Preprocessing

In machine learning-based modeling, the input variables are inevitably subject to significant dimensional heterogeneity in both measurement units and statistical distributions. When directly introduced into the training process, variables with dominant numerical magnitudes, such as conductivity, may exert disproportionate leverage on gradient descent and parameter estimation, whereas those with smaller ranges, such as temperature, may be computationally marginalized. To mitigate scale-dependent bias and ensure feature parity, all input variables underwent min–max normalization prior to model architecture development, mapping them to a comparable numerical scale. This preprocessing procedure was adopted to reduce the interference caused by magnitude heterogeneity among variables and to enhance training stability, convergence behavior, and the model’s generalization capabilities.

Although feed intake is generally associated with fish population size within a production unit, appetitive dynamics are also modulated by ontogenetic development and ambient environmental conditions. Accordingly, prior to model construction, the relationships among feeding behavior, biological variables, environmental factors, and actual population size were examined to determine the relative contributions to feeding dynamics [19]. In this study, Pearson correlation analysis was conducted on the FSSP and FFIF datasets to evaluate the linear associations among the 16 input variables and the target output variable. The resulting correlation matrix quantitatively characterized the links among feed intake, environmental and biological factors, and fish population size, thereby providing an analytical basis for subsequent feature engineering and the mechanistic interpretation of predictive outcomes.

After data normalization and correlation analysis, the dataset was randomly partitioned into training (80%) and testing (20%) subsets. The training subset was used for model construction and parameter optimization, whereas the testing subset was reserved exclusively for the final performance evaluation.

Data normalization formula:

(3) $$X_{i}\;=\;\frac{x_{i}-x_{\min}}{x_{\max}-x_{\min}}$$

where $X_{i}$ is the normalized feature value, $x_{i}$ is the original observation, $x_{\min}$ is the minimum value of the specific feature, $x_{\max}$ is the maximum value of the feature recorded in the dataset.

Pearson correlation coefficient formula:

(4) $$r_{\mathrm{xy}}\;=\;\frac{\sum_{i=1}^{n}\left(x_{i}-\bar{x}\right)\left(y_{i}-\bar{y}\right)}{\sqrt{\sum_{i=1}^{n}{\left(x_{i}-\bar{x}\right)}^{2}}\cdot \sqrt{\sum_{i=1}^{n}{\left(y_{i}-\bar{y}\right)}^{2}}}$$

where $r_{\mathrm{xy}}$ is the Pearson correlation coefficient between input variables x and output y, $x_{i}$ is the value of variable x in the $i^{\mathrm{th}}$ sample, $y_{i}$ is the value of variable y in the $i^{\mathrm{th}}$ sample, $\bar{x}$ is the sample mean of variable x, $\bar{y}$ is the sample mean of variable y, n is the sample size.

### 2.4. Standard RBF Neural Network

The standard radial basis function (St-RBF) architecture was adopted in this study due to its strong nonlinear approximation capabilities and computational tractability. The structural configuration of the St-RBF network features a tri-layer topology: an input layer, a hidden layer, and an output layer. The input layer receives multidimensional vectors including daily feed ration, water temperature, body weight, age (days post-hatch), conductivity, and other related covariates. The hidden layer projects the input variables into a higher-dimensional space through radial basis functions, facilitating the extraction and characterization of complex, local nonlinear manifolds within the data. Finally, the output layer generates the population size through a weighted linear combination of the hidden-layer activations. The fundamental procedures involved in the development and parameterization of the St-RBF model are presented below. The St-RBF model was implemented as a conventional baseline model, and no additional optimization or regularization techniques were applied to its hidden-layer centers, width parameters, or output-layer weights. All subsequent optimization procedures were performed within the RBF framework to evaluate the effects of center-width optimization, output-layer optimization, activation-function selection, and bioenergetic feature embedding under a unified model structure.

(1) Determination of hidden-layer parameters

The K-means clustering algorithm was employed to partition the training dataset comprising multidimensional covariates into K clusters. The centroid of each cluster was then designated as the center vector for the corresponding hidden neuron within the RBF network.

(5) $$J=\sum_{i=1}^{K}\;\sum_{x\in C_{i}}\;∥x-c_{i}{∥}^{2}$$

where J is the objective of the clustering algorithm, representing the minimization of the sum of squared errors within clusters, $c_{i}$ is the center vector of the i-th hidden layer node, K is the number of hidden layer nodes (i.e., the number of clusters), x is the input vector of the training sample, $C_{i}$ is the sample set belonging to the i-th cluster.

Upon determining the center vectors, the pairwise distances between cluster centers were further calculated to determine the corresponding width parameters of the hidden layer.

(6) $$\sigma_{i}=\frac{1}{K}\sum_{j=1}^{K}\;∥c_{i}-c_{j}∥$$

where $\sigma_{i}$ is the width parameter of the i-th hidden layer node, $∥c_{i}-c_{j}∥$ is the Euclidean distance between the center vectors $c_{i}$ and $c_{j}$, K is the number of hidden layer nodes.

(2) Determination of output-layer weights

The response of each hidden-layer neuron to the input vector was calculated using the radial basis function, and the corresponding hidden-layer output matrix was subsequently constructed for all training samples. Once the target matrix was constructed from the desired outputs of the training dataset, ordinary least squares linear regression was applied to compute the output-layer weight coefficients.

(7) $$h_{i}\left(x\right)=\exp\left(-\frac{∥x-c_{i}{∥}^{2}}{2\sigma_{i}^{2}}\right)$$

where $h_{i}\left(x\right)$ is the response of the i-th hidden layer node to the input vector x, x is the input vector, $c_{i}$ is the center vector of the i-th hidden layer node, $\sigma_{i}$ is the width parameter of the i-th hidden layer node.

(8) $$H_{\mathrm{ji}}=h_{i}(x_{j})$$

where H is the hidden layer output matrix, $x_{j}$ is the input vector of the j-th training sample, $h_{i}(x_{j})$ is the response of the i-th hidden layer node to the j-th training sample.

(9) $$W=(H^{T}H{)}^{-1}H^{T}Y$$

where W is the output layer weight matrix, H is the hidden layer output matrix, and Y is the target output matrix.

### 2.5. Optimization of the RBF Neural Network

#### 2.5.1. Center and Width Optimization of the RBF Neural Network Based on PSO

Particle Swarm Optimization (PSO) is a population-based optimization algorithm that searches for the global optimum through the cooperative movement of particles within the solution space [20]. Due to its robust convergence characteristics and low computational complexity, PSO has been extensively applied in parameter optimization [20]. Within the PSO framework, each particle represents a candidate solution and is defined by its position and velocity. The particle swarm is randomly initialized within the search space, and the fitness of each particle is evaluated using a predefined objective function. Throughout the iterative search process, each particle updates its trajectory based on both its personal best position (pBest) and the global best position (gBest) identified by the swarm. Specifically, a particle’s velocity is adjusted using an inertia weight component together with two acceleration terms associated with pBest and gBest, respectively, and, subsequently, its position is updated based on the newly calculated velocity. This optimization procedure is repeated until a stopping criterion is met, such as reaching a maximum number of iterations or attaining a predefined fitness threshold. The final gBest obtained upon convergence represents the optimal solution. In the present study, PSO was employed to optimize the centers and widths of the RBF network. The primary implementation steps of the PSO-RBF model are summarized as follows.

(1) Particle swarm initialization

The particle swarm was randomly initialized within the search space, with each particle encoding a candidate parameter set comprising the center vectors and width parameters of the RBF network. The initial position and velocity of each particle were assigned randomly, representing the current candidate solution and its corresponding search trajectory, respectively.

(2) Fitness evaluation

The root mean square error (RMSE) calculated on the training dataset was employed as the fitness function to evaluate the quality of each particle throughout the PSO optimization process. The testing dataset was excluded from the optimization procedure and was used only for the final performance evaluation of the optimized model. The fitness value of each candidate solution was subsequently calculated based on the model’s prediction error.

(3) Iterative optimization and convergence

Particle positions and velocities were iteratively updated until a stopping criterion was met, such as reaching the maximum number of iterations or attaining a predefined fitness threshold.

Velocity update equation:

(10) $$v_{i}(t\;+\;1)\;=\;w\cdot v_{i}\left(t\right)\;+\;c_{1}\cdot r_{1}\cdot (\mathrm{pBes}t_{i}-x_{i}\left(t)\right)\;+\;c_{2}\cdot r_{2}\cdot (\mathrm{gBest}-x_{i}\left(t)\right)$$

Position update equation:

(11) $$x_{i}(t\;+\;1)\;=\;x_{i}\left(t\right)\;+\;v_{i}(t\;+\;1)$$

where $v_{i}\left(t\right)$ is the velocity of particle i at time t, $x_{i}\left(t\right)$ is the position of particle i at time t, w is the inertia weight, $c_{1}$ and $c_{2}$ are learning factors, $r_{1}$ and $r_{2}$ are random numbers in the range [0, 1], $\mathrm{pBes}t_{i}$ is the personal best position of particle i, gBest is the global best position.

(4) Determination of optimal parameters

Upon completion of the iterative search process, the global best position (gBest) identified by the swarm was adopted as the optimal parameter configuration for subsequent network construction.

(5) RBF neural network training

The optimized center vectors and width parameters were incorporated into the RBF network to initialize the hidden layer, after which the network was trained using the designated training dataset.

#### 2.5.2. Output-Layer Optimization of the RBF Neural Network Based on Ridge Regression

In standard RBF networks, the output-layer weight coefficients are typically determined using the ordinary least squares (OLS) method, which estimates the weights by minimizing the sum of squared errors between the predicted and observed values [21]. Despite its computational simplicity and efficiency, this method can become unstable when the hidden-layer output matrix is ill-conditioned, thereby increasing the risk of overfitting and compromising the model’s generalization capabilities.

To address this limitation, ridge regression was employed in the present study for output-layer optimization. By introducing an L_2_ regularization term into the objective function, ridge regression constrains the magnitude of the weight coefficients, thereby reducing estimation variance and enhancing the model’s robustness against noise. The corresponding objective function is formulated as follows:

(12) $$w\;=\;{\left(H^{T}H\;+\;\lambda I\right)}^{-1}H^{T}y$$

where w is the weight vector of the output layer, H is the hidden layer output matrix, $H^{T}$ is the transpose of H, λ is the regularization coefficient, I is the identity matrix, y is the actual output value vector.

This optimization strategy enhances the numerical stability of weight estimation, particularly when the hidden layer contains a large number of neurons or when multicollinearity exists among the hidden-layer features. Additionally, the predictive performance of the OLS method was systematically compared with that of ridge regression to further evaluate their respective impacts on model accuracy and stability.

#### 2.5.3. Activation-Function Optimization of the RBF Neural Network Based on the Inverse Quadratic Function

The activation function is a critical determinant of the predictive performance of RBF networks [22]. In conventional RBF models, the Gaussian function is predominantly employed due to its localized response property, making it generally suitable for relatively smooth data distributions [22]. However, when input variables exhibit outliers or heavy-tailed distributions, the Gaussian response decays rapidly with increasing distance from the center; this can diminish the contribution of distant yet informative samples, consequently compromising model robustness and generalization capabilities [23].

To address this limitation, the inverse quadratic function was introduced in the present study as an alternative radial basis function. Compared with the Gaussian function, the inverse quadratic function exhibits a more gradual decay pattern away from the center, thereby maintaining a broader response range and heightened sensitivity to distant samples. This characteristic is highly advantageous for capturing heterogeneous feature distributions and enhancing model adaptability under complex data conditions. Its mathematical expression is formulated as follows:

(13) $$\phi \left(x\right)\;=\;\frac{1}{∥x-c{∥}^{2}\;+\;\sigma^{2}}$$

where x is the input vector, c is the center vector, σ is the width parameter, and ∥x−c∥ is the Euclidean distance.

To systematically evaluate the impact of activation function selection on model performance, two RBF-based predictive models were constructed within a unified computational framework using the Gaussian and inverse quadratic functions, respectively. Their predictive performance was subsequently compared using MAE, MAPE, and RMSE, thereby providing an empirical basis for activation-function selection in subsequent modeling stages.

#### 2.5.4. RBF Neural Network Optimization Method Based on GA

The genetic algorithm (GA) is a population-based optimization technique characterized by robust global search capabilities, widely used to mitigate the risk of premature convergence to local optima within complex solution spaces [24]. In the present study, the GA was employed to optimize the parameters of the RBF neural network under the same architectural configuration as that adopted in the PSO-based model. The predictive performance of the GA-RBF model was subsequently compared with that of the PSO-RBF model to evaluate their relative optimization efficacy. This comparison provided an empirical basis for selecting the most appropriate optimization strategy for subsequent modeling. The detailed implementation procedures of the GA-RBF framework followed those outlined in previous studies [6,25,26].

### 2.6. Embedding Bioenergetic Principles of Large Yellow Croaker into the Improved RBF Neural Network

To further enhance the predictive performance and biological relevance of the proposed fish population estimation model, the bioenergetic characteristics of the large yellow croaker were embedded into the improved RBF neural network. Specifically, variables related to energy acquisition, energy expenditure, and metabolic balance were incorporated into the model’s input structure, thereby enabling the prediction of fish populations to explicitly account for the species-specific physiological mechanisms underlying feeding regulation and growth. These bioenergetic variables were incorporated as mechanism-derived descriptors of energy acquisition, energy expenditure, and growth processes, rather than as independently fitted variables.

The corresponding bioenergetic equations are formulated as follows:

(14) F = α·C

(15) $$U=\beta \cdot \left(C-F\right)$$

(16) $$R=\gamma \cdot W^{\delta }\cdot e^{\theta T}$$

(17) G=C−R−F−U

where F is the fecal output, α is the fecal conversion coefficient, C is the daily feed ration, U is the excretory output, β is the excretion conversion rate, R is the basal metabolic consumption, W is the body weight, T is the water temperature, γ, δ, and θ are parameter constants, and G is the energy growth. Daily feed ration and water temperature were obtained directly from the experimental records. The parameter constants used in the bioenergetic equations were adopted from previously published bioenergetic studies on large yellow croaker and related marine teleost species [27] and were kept fixed throughout model training and evaluation.

Body weight was measured weekly throughout the experiment, whereas daily feed ration and water temperature were recorded throughout the feeding trial. The weekly mean body weight was used in the bioenergetic calculations for the corresponding week, while fish population size was updated every two days according to the actual fish removal records. Thus, each variable was incorporated into the model according to its corresponding sampling frequency.

### 2.7. Evaluation Metrics

To evaluate the predictive performance of different modeling strategies, mean absolute error (MAE), mean absolute percentage error (MAPE), root mean square error (RMSE), relative error (RE), and absolute error (AE) were employed as the principal evaluation metrics in the present study. The corresponding mathematical expressions are formulated as follows:

(18) $$\mathrm{MAE}\;=\frac{\Sigma_{i=1}^{T}|Y_{i}-y_{i}|}{T}\;$$

(19) $$\mathrm{MAPE}=\sum_{i=1}^{T}\;\left|\frac{Y_{i}-y_{i}}{Y_{i}}\right|\frac{100}{T}$$

(20) $$\mathrm{RMSE}=\sqrt{\frac{1}{T}\sum_{i=1}^{T}\;(Y_{i}-y_{i}{)}^{2}}$$

(21) $${\mathrm{RE}}_{i}=\frac{y_{i}-Y_{i}}{Y_{i}}\times 100\%$$

(22) $${\mathrm{AE}}_{i}=\left|{Y_{i}-y}_{i}\right|$$

$Y_{i}$ is the actual value of fish school quantity, $y_{i}$ is the predicted value of fish school quantity, T is the total number of samples.

## 3. Results

### 3.1. Comprehensive Screening of Factors Influencing Fish Feeding Behavior and Population Size

**Figure 1:**
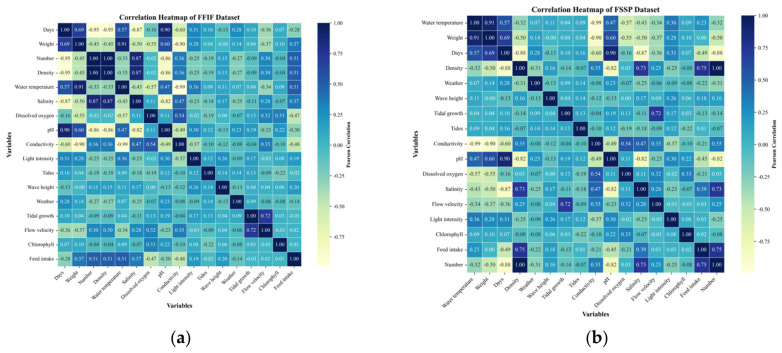
Pearson correlation heatmaps between input and output variables under different dataset conditions. This figure illustrates the Pearson correlation between model input variables and output targets under two datasets. Cooler colors indicate stronger positive correlations, while warmer colors represent stronger negative correlations. Subfigure (**a**) uses fish feed intake as the target variable, while Subfigure (**b**) focuses on fish school count.

To identify the primary factors associated with feeding behavior and fish population size, Pearson correlation analysis was conducted on the FFIF and FSSP datasets. In the FFIF dataset (Figure 1a), water temperature (r = 0.51), body weight (r = 0.37), age (r = −0.28), stocking density (r = 0.51), stocking number (r = 0.51), conductivity (r = −0.48), pH (r = −0.30), dissolved oxygen (r = −0.47), and salinity (r = 0.37) exhibited notable correlations with feed intake. In contrast, weather, wave height, and light intensity demonstrated weaker correlations with feed intake. In the FSSP dataset (Figure 1b), most variables demonstrated varying degrees of correlation with fish population size, whereas tide, dissolved oxygen, and chlorophyll concentration exhibited comparatively weak associations.

### 3.2. Prediction Performance of the Standard RBF Neural Network

The primary parameters of the St-RBF model are listed in Table 2, and representative prediction results for the test subset are summarized in Table 3. Model performance differed substantially across samples. Specifically, the RE for Sample 3 was 2.61%, whereas Sample 6 exhibited a markedly larger deviation, with an RE of 93.56%. The AE ranged from 25 to 741. For the entire test set, the MAE, MAPE, and RMSE reached 278.38, 33.93%, and 380.06, respectively.

**Figure 2:**
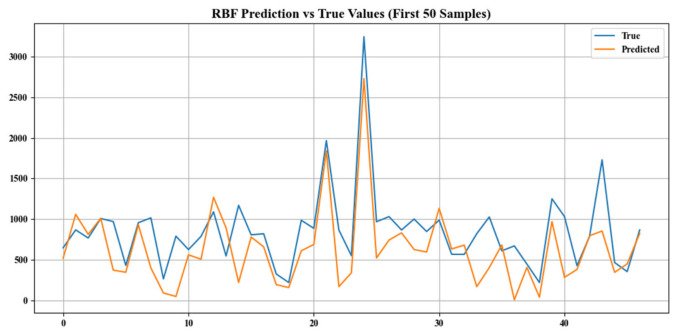
Comparison of predicted and actual fish school quantities using the St-RBF model (first 50 samples).

As shown in Figure 2, the predicted and observed values for the first 50 samples exhibited generally consistent trends. Nevertheless, larger deviations were observed during periods of abrupt fluctuations and in several extreme cases.

**Figure 3:**
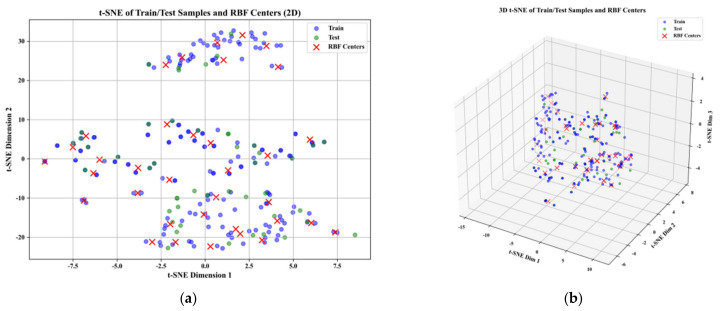
Visualization of St-RBF model center distributions based on t-SNE dimensionality reduction. (**a**) Two-dimensional projection of the same RBF centers, showing their spatial dispersion relative to input data points. (**b**) Three-dimensional distribution of RBF centers, training and test samples after t-SNE mapping.

Figure 3 presents the distribution of RBF centers in the feature space following t-SNE dimensionality reduction, visualized in both 2D and 3D projections. The projected centers were relatively evenly distributed and showed no pronounced clustering within a limited region, effectively covering most of the areas occupied by the training and test samples.

### 3.3. Impact of PSO-Based Optimization on the Predictive Performance of the RBF Neural Network

#### 3.3.1. Impact of PSO-Based Optimization of Centers, Widths, and Weights on the Predictive Performance of the RBF Neural Network

**Figure 4:**
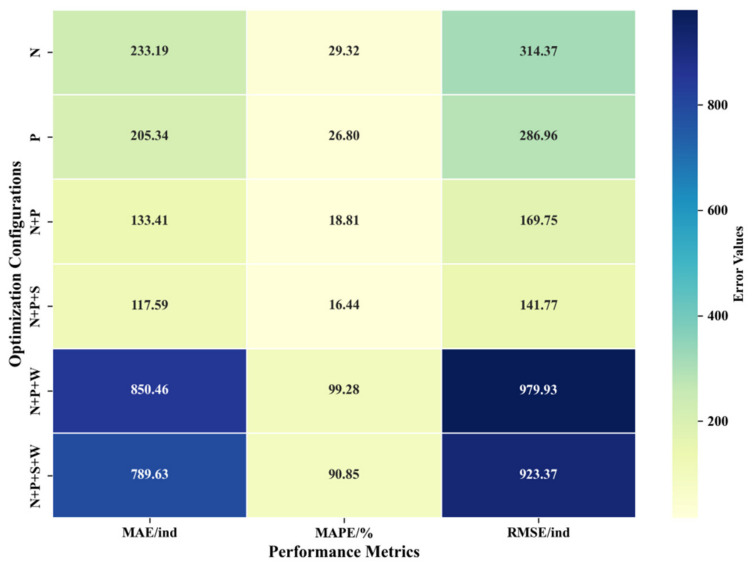
Performance comparison of RBF model under different optimization configurations. N = number of RBF centers; P = positions of RBF centers; S = spread (width) parameters of RBF nodes (σ); W = output weights connecting hidden layer to the output layer.

As shown in Figure 4, different PSO-based optimization strategies yielded markedly different effects on the predictive performance of the RBF network. Optimization of the number of centers (N) or center positions (P) alone reduced prediction error, yielding MAE values of 233.19 and 205.34, and MAPE values of 29.32% and 26.80%, respectively. The simultaneous optimization of N and P further reduced the prediction error (MAE = 133.41, MAPE = 18.81%). Incorporating the width parameters into the optimization framework (N + P + S) yielded the optimal predictive performance, achieving MAE, MAPE, and RMSE values of 117.59, 16.44%, and 141.77, respectively. Conversely, when the output-layer weights were integrated into the joint optimization schemes (N + P + W or N + P + S + W), the model’s performance deteriorated substantially, as evidenced by pronounced increases in both the MAE and MAPE.

**Figure 5:**
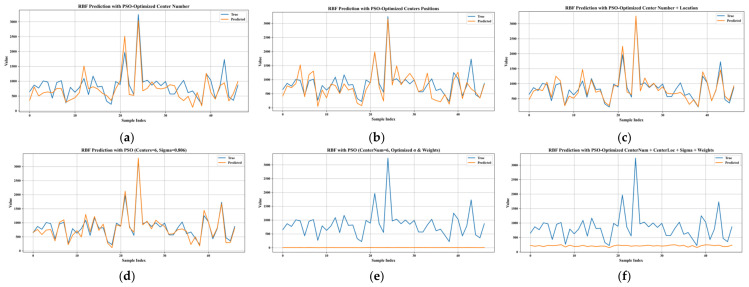
Comparison of prediction performance of RBF models under different PSO optimization configurations. (**a**) Prediction curve with PSO-optimized center number; (**b**) center position; (**c**) center number and position; (**d**) center number, position, and spread; (**e**) center number, position, and output weights; (**f**) full optimization including center number, position, spread, and weights.

As illustrated in Figure 5, the prediction curves generated under different PSO optimization configurations exhibited distinct fitting characteristics. When only the number or positions of the centers were optimized (Figure 5a,b), the predicted curves generally tracked the trends of the observed values; however, the overall fitting precision remained limited. The joint optimization of N and P (Figure 5c) noticeably improved the alignment between the predicted and observed curves, particularly in regions characterized by pronounced peaks and abrupt fluctuations. The further inclusion of width optimization (Figure 5d) resulted in a tighter overall fit and minimized error fluctuations. In contrast, when the output-layer weights were jointly optimized (Figure 5e,f), the predictive performance was markedly impaired, with certain predicted curves flattening into near-constant values or deviating substantially from the observed data.

#### 3.3.2. Effect of Output-Layer Optimization Methods on the Predictive Performance of the RBF Neural Network

**Figure 6:**
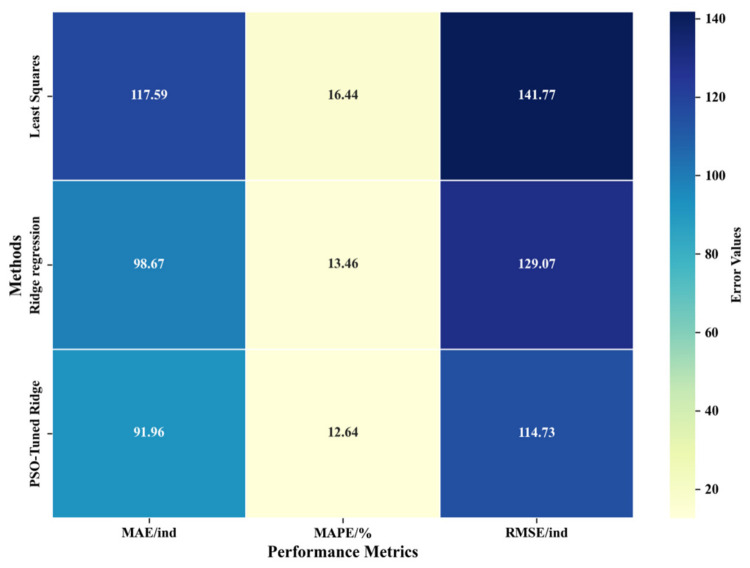
Comparison of output weight solving methods for RBF models. PSO-tuned ridge indicates ridge regression with the regularization parameter λ tuned via particle swarm optimization (PSO).

**Figure 7:**
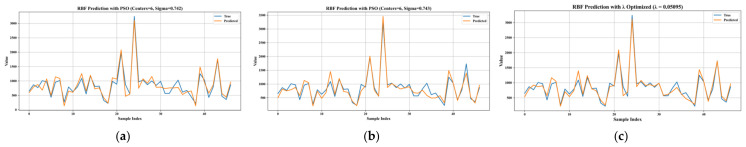
Comparison of prediction performance of RBF models with different output weight solving methods. (**a**) Prediction curve of the RBF model using Gaussian kernel; (**b**) prediction curve of the RBF model with output weights solved via ridge regression (λ = 0.8099); (**c**) prediction curve of the RBF model with λ optimized by PSO (λ = 0.4392).

As shown in Figure 6, when the OLS method was employed to determine the output-layer weights, the model exhibited relatively limited predictive accuracy, with MAE, MAPE, and RMSE values of 117.59, 16.44%, and 141.77, respectively. In contrast, applying ridge regression further enhanced model performance, reducing the MAE, MAPE, and RMSE to 98.67, 13.46%, and 129.07, respectively. The subsequent optimization of the regularization coefficient λ via PSO yielded additional performance gains, with the corresponding error metrics further decreasing to 91.96, 12.64%, and 114.73, respectively. As illustrated in Figure 7, the optimized model exhibited a superior overall fit and greater predictive stability.

#### 3.3.3. Impact of Different Activation Functions on the Predictive Performance of the RBF Neural Network

**Figure 8:**
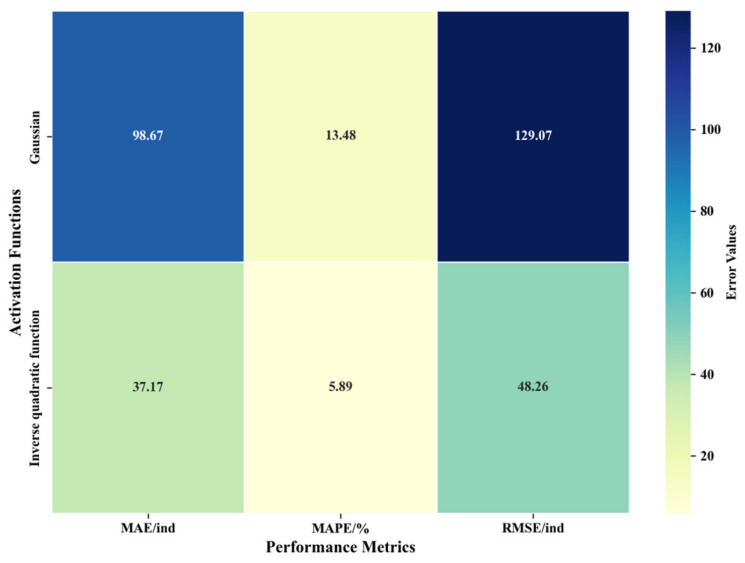
Effects of different activation functions on the prediction performance of the RBF models.

**Figure 9:**
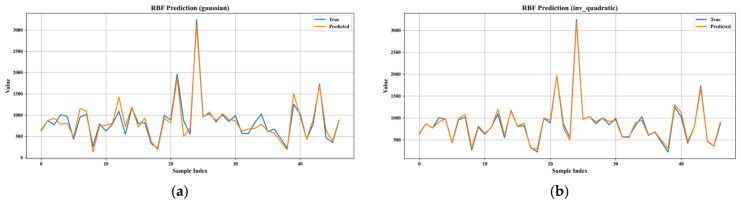
Effects of different activation functions on the prediction performance of the RBF models. (**a**) RBF model based on the Gaussian function; (**b**) RBF model based on the inverse quadratic function.

As shown in Figure 8, the choice of activation function markedly influenced the predictive performance of the RBF model. When the Gaussian function was adopted, the MAE, MAPE, and RMSE were calculated as 98.67, 13.48%, and 129.07, respectively. In contrast, implementing the inverse quadratic function substantially reduced the prediction error, with the corresponding MAE, MAPE, and RMSE decreasing to 37.17, 5.89%, and 48.26, respectively. This improvement is further corroborated by the prediction curves illustrated in Figure 9. In regions where the fish population size changed abruptly, the Gaussian-based model exhibited pronounced deviations from the observed values. By comparison, the model utilizing the inverse quadratic function more accurately captured the actual trends, particularly in high-value intervals where the fitted curves remained highly stable.

### 3.4. Impact of Different Optimization Algorithms on Predictive Performance

**Figure 10:**
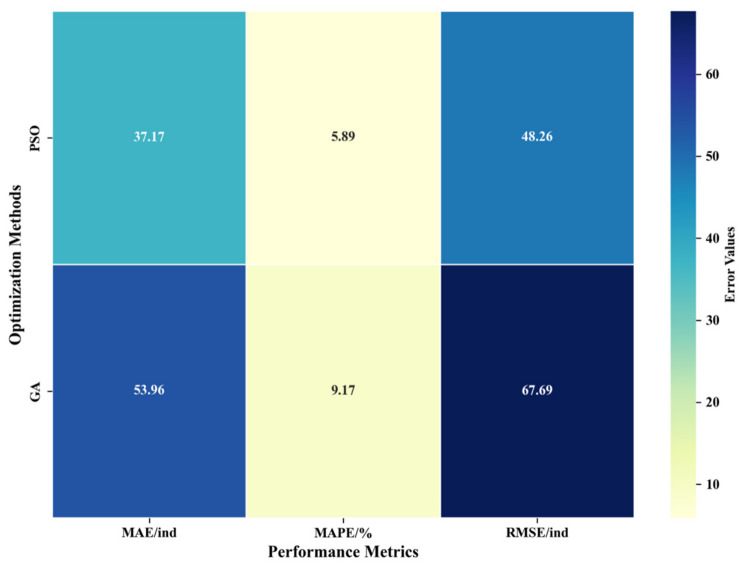
Effects of different optimization algorithms on the prediction performance of RBF models.

**Figure 11:**
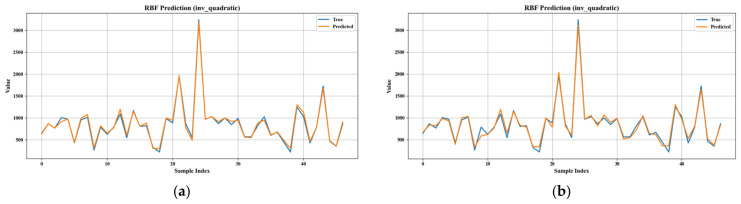
Comparison of prediction performance of RBF models under different optimization algorithms. (**a**) Prediction curve of the RBF model optimized by PSO; (**b**) prediction curve of the RBF model optimized by GA.

As illustrated in Figure 10, the PSO-optimized model achieved superior predictive performance compared to the GA-optimized model, yielding MAE, MAPE, and RMSE values of 37.17, 5.89%, and 48.26, respectively. In comparison, optimization via the GA resulted in higher prediction errors, with the corresponding MAE, MAPE, and RMSE increasing to 53.96, 9.17%, and 67.69, respectively. As further illustrated in Figure 11, the prediction curves obtained under PSO optimization were generally closer to the observed values and more accurately reflected the underlying trends of the fish population size. By contrast, under GA optimization, noticeable deviations between the predicted and observed values persisted across several intervals.

### 3.5. Incorporation of the Bioenergetic Principles of Large Yellow Croaker into the Enhanced RBF Neural Network

**Figure 12:**
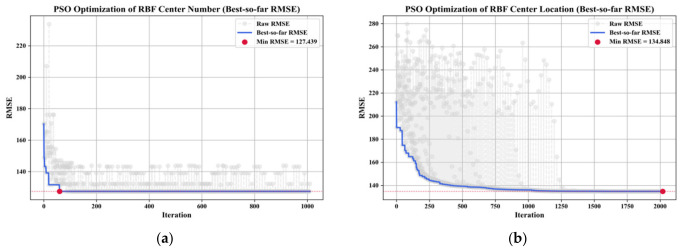

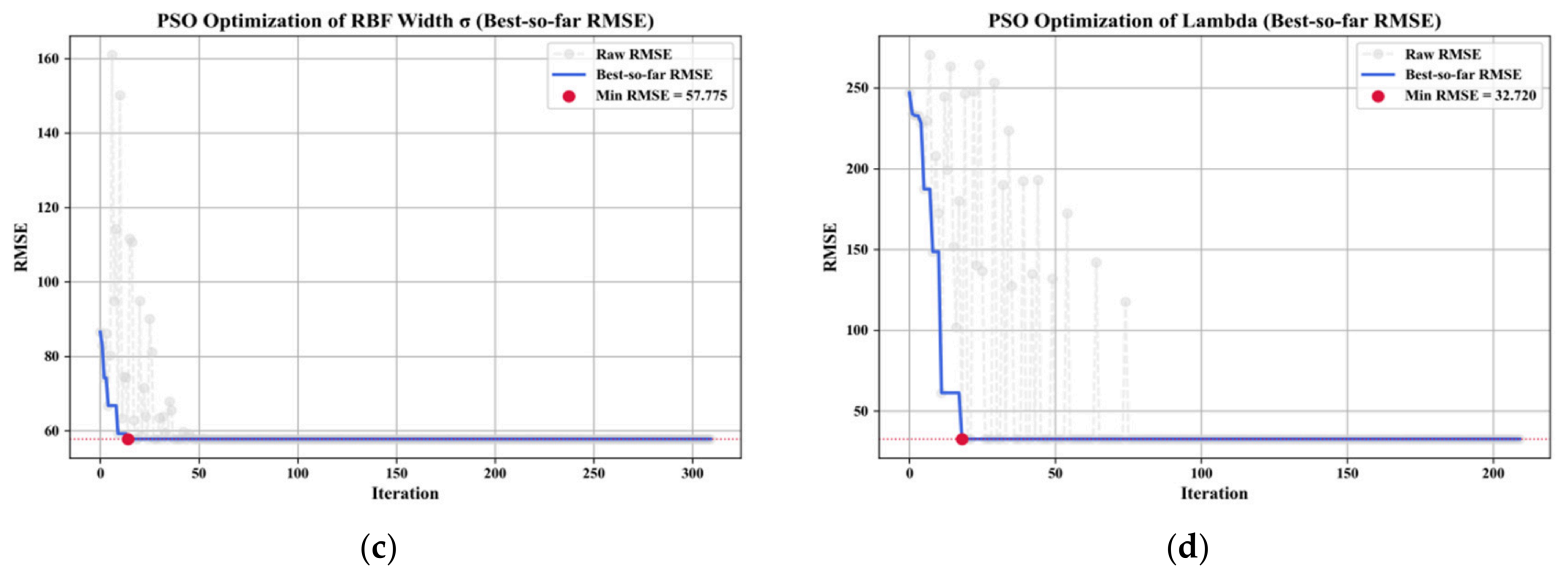
PSO-based optimization of hyperparameters for the BE-RBF neural network. RMSE convergence trends during PSO optimization of the four key hyperparameters of the bioenergetics-enhanced RBF neural network: (**a**) number of centers, (**b**) center locations, (**c**) kernel width σ, and (**d**) regularization parameter λ. The RMSE values represent the model’s performance on the test set at each PSO iteration, recorded in real time during the optimization process.

**Figure 13:**
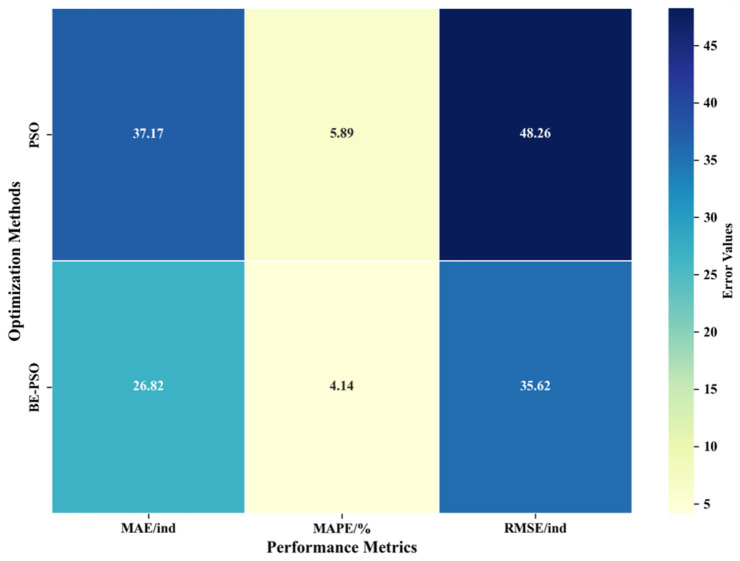
Effects of integrating bioenergetics principles into the PSO-RBF model on the prediction performance for large yellow croaker.

**Figure 14:**
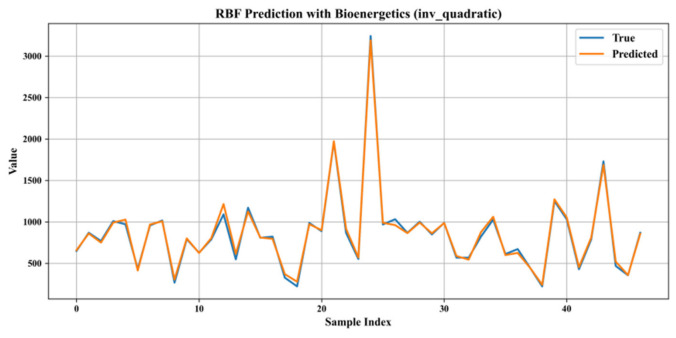
Prediction performance of RBF model optimized with bioenergetics-based input features.

The bioenergetic parameters of the large yellow croaker are listed in Table 4. As depicted in Figure 12, the key parameters of the model converged within a limited number of iterations. Compared with the conventional PSO-RBF model, the BE-PSO-RBF model further reduced prediction errors following the incorporation of the bioenergetic mechanism. The resulting MAE, MAPE, and RMSE were 26.82, 4.14%, and 35.62, respectively, representing reductions of 27.9%, 29.7%, and 26.2% relative to the original model (Figure 13). As illustrated in Figure 14, the predicted curve closely tracked the observed values. Robust fitting performance was maintained across samples of varying magnitudes, ensuring highly stable overall prediction results.

**Figure 15:**
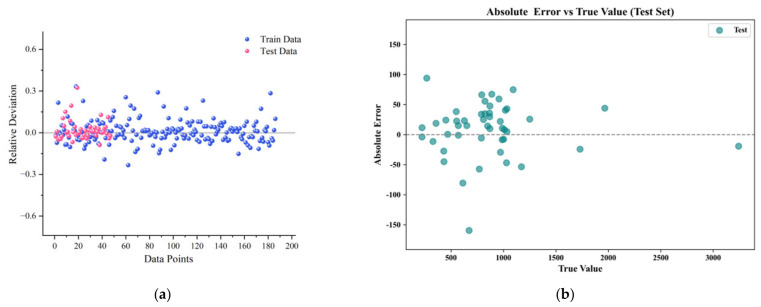
Error analysis of BE-PSO-RBF model prediction results. (**a**) Relative deviation distribution of training and test samples, used to assess the consistency of model predictions and the fluctuation of deviation across different data points; (**b**) scatter plot of absolute prediction error versus true value on the test set, used to observe the variation in prediction error corresponding to different target values.

Figure 15 presents the error distribution of the BE-PSO-RBF model across the training and testing phases. The majority of samples exhibited only minimal deviations. In the training set, 55.91% of the samples yielded an RE within ±0.05, whereas the corresponding proportion in the testing set reached 80.85%. For an RE threshold of ±0.10, these proportions increased to 82.80% and 87.23%, respectively (Table 5). The AE was predominantly concentrated within ±100 individuals, with 82.98% of the samples demonstrating an AE of fewer than 50 individuals (Table 6). Even for samples with observed populations exceeding 1500 and approaching 3000 individuals, the prediction error remained consistently within ±100 individuals.

**Figure 16:**
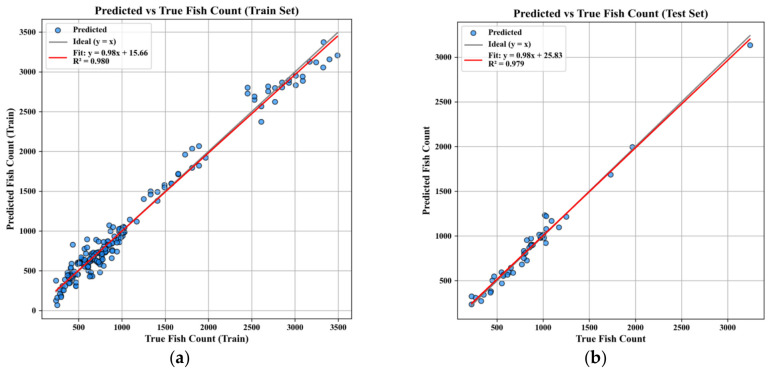
Regression performance of the BE-PSO-RBF model on training and test sets. (**a**) Regression between predicted and true fish counts in the training set; (**b**) fit of prediction results to true values in the test set. The red line denotes the linear fit, and the gray dashed line represents the ideal y = x line.

**Figure 17:**
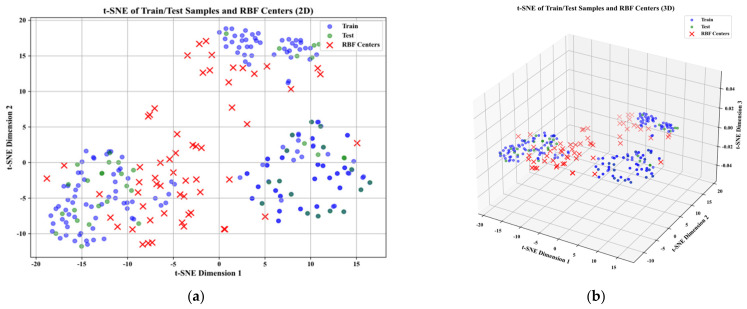
BE-PSO-RBF model center distribution in embedded space. (**a**) Two-dimensional projection showing the distribution pattern and coverage of centers relative to the input space. (**b**) Three-dimensional distribution of improved RBF centers after t-SNE mapping, reflecting spatial relationships with data samples.

As presented in Figure 16, the coefficients of determination (R^2^) reached 0.980 and 0.979 for the training and testing sets, respectively, with the majority of prediction points tightly clustered along the 1:1 line. Figure 17 further illustrates the distribution of the BE-PSO-RBF centers following dimensionality reduction. While the centers were generally situated within the principal sample regions, their coverage was not entirely uniform, leaving certain local clusters underrepresented.

## 4. Discussion

Unlike direct counting approaches based on optical or acoustic sensing, the present study explored whether feeding-derived information could be used as an indirect biological proxy for estimating fish population size in cage aquaculture. Although some optical- and acoustic-based fish counting methods have reported slightly higher counting accuracies under specific experimental or image acquisition conditions, direct comparison with the present study should be interpreted with caution. Most existing approaches estimate fish numbers through visual or acoustic detection of individual fish or density maps, and their performance is strongly dependent on image quality, water transparency, fish occlusion, background contrast, and sensor coverage [5,6,7,8,9,10,11,12]. In contrast, the present study did not aim to replace high-resolution sensing-based counting under controlled conditions, but to develop an alternative indirect estimation framework for open cage aquaculture, where continuous and reliable visual or acoustic acquisition is often difficult. The slightly lower accuracy of the proposed model may be partly attributed to the indirect nature of feeding-based estimation, because feed intake is influenced not only by population size but also by environmental fluctuations, feeding behavior, body size, and physiological status. Nevertheless, the BE-PSO-RBF model indicates that feeding-derived information, when integrated with biological variables, environmental parameters, and bioenergetic features, can serve as a biologically meaningful proxy for population estimation. Therefore, although the estimation accuracy of the proposed model was slightly lower than that reported in some image-based studies, this approach offers advantages in terms of operational feasibility, low dependence on underwater visibility, and potential scalability in practical cage farming scenarios.

t-SNE visualization of the model’s structural optimization reveals that the center distribution of the St-RBF model is relatively uniform in both two- and three-dimensional projections, covering the majority of data samples. However, following PSO optimization, the uniformity of RBF centers in low-dimensional projections decreases, with certain regions exhibiting pronounced sparsity. This phenomenon can be attributed to the objective function of PSO, which prioritizes the minimization of prediction errors over maintaining a visually uniform center distribution in low-dimensional projections [28,29,30]. Essentially, PSO enhances predictive performance through the strategic placement of centers within the high-dimensional space, regardless of their visual uniformity in low-dimensional embeddings [31,32]. In the context of data analysis, Sheikhan et al. observed that t-SNE visualizations can suffer from dimensionality reduction distortion, often failing to accurately preserve the underlying semantic structure of high-dimensional data. To prevent evaluation bias, they emphasized that visual uniformity should not serve as the sole criterion for assessing the integrity of network structures [33]. Similarly, when analyzing node distributions in PCA-based RBF networks, Zhang et al. reported potential discrepancies between visual clustering patterns and actual model performance [34]. Consistent with these observations, this study further distinguishes between low-dimensional visual representations and actual model efficacy, emphasizing that the primary evaluation metric must be the model’s performance in the high-dimensional feature space. This underscores the importance of evaluating models within their native high-dimensional domains and cautions against relying exclusively on dimensionality reduction projections when assessing the effectiveness of RBF neural networks.

It is worth noting that when employing PSO to optimize output weights (e.g., the N + P + W and N + P + S + W configurations), the predictive performance of the model deteriorates significantly. Initially, this observation appears to contradict the intuitive expectation that optimizing a larger number of parameters inherently yields superior accuracy [35]. Rather, this phenomenon reflects the inherent asymmetry of parameter characteristics across different layers within the RBF architecture and the incompatibility of specific optimization strategies [36]. In the classic RBF framework, the estimation of output weights is formulated as a standard linear regression problem, which can be efficiently resolved using established analytical methods such as ordinary least squares [37,38]. Previous studies have emphasized that this linearity is a critical factor in maintaining the simplicity and stability of RBF networks. When the stochastic global search strategy of PSO is directly applied to output weights, its black-box and gradient-free nature predisposes the algorithm to becoming trapped in local optima within the high-dimensional weight space; this causes overfitting to a limited number of outliers and compromises the global fitting capability and generalization performance inherently provided by analytical linear solutions [39,40]. Furthermore, the dimensionality of the output weights is typically substantially higher than that of the center positions or width parameters. In a multi-input, multi-center configuration, this high dimensionality leads to a severe decline in the search efficiency of the PSO algorithm and reduces convergence reliability. Similar phenomena have been documented in studies concerning high-dimensional particle optimization. The literature indicates that when the parameter space exceeds 50 dimensions, PSO suffers from substantial search degradation and convergence oscillation [41,42]. From a methodological perspective, integrating output weights into a PSO-based joint optimization framework not only lacks structural compatibility but also introduces supplementary instability, thereby degrading overall performance [43,44]. In this study, ridge regression is employed as the core analytical strategy for the output layer, coupled with PSO to optimize the regularization parameter λ. This approach achieves simultaneous enhancements in prediction accuracy and model stability without inflating computational complexity. These improvements are attributable to the regularization term within the loss function, which effectively alleviates weight divergence issues induced by sample noise or feature collinearity, thereby fostering robust and stable overall model performance [45,46].

Even after optimizing the structural parameters, the choice of activation function remains a critical determinant of RBF neural networks. In this study, substituting the traditional Gaussian kernel with the inverse quadratic function significantly enhances both nonlinear fitting capabilities and error control. Compared to the Gaussian kernel, the inverse quadratic function exhibits a more gradual decay and a broader receptive field near the center, which demonstrates distinct advantages in complex environments characterized by substantial variations in fish population size, particularly when responding to regions with severe feature fluctuations or high sample density. These observations align with findings from previous studies [47,48]. Existing literature indicates that alternative kernel functions can outperform Gaussian kernels in specific tasks. For instance, the Cauchy kernel has demonstrated advantages in resolving sharp boundary problems, whereas polynomial kernels have proven more effective at capturing long-range nonlinear correlations within complex datasets [49,50]. These findings suggest that although Gaussian kernels are ubiquitous, they are not universally optimal. The results of this study expand upon current understanding by demonstrating that non-Gaussian radial basis functions, such as the inverse quadratic function, possess superior adaptability and generalization capabilities. Prior research emphasizes that the selection of an activation function should be congruent with the distribution characteristics of the target variable. Furthermore, it is well-established that the response width of a kernel function plays a critical role in determining its coverage of the input space. Additionally, kernels with slower decay characteristics can enhance the stability of model fitting in regions characterized by high sample density and low noise levels [51,52]. This premise is corroborated by the present study, wherein the inverse quadratic kernel achieves smoother and more accurate fitting performance across ranges of high fish density. Ultimately, these results underscore that the activation function is not an arbitrarily selected hyperparameter. Rather, its selection requires a comprehensive evaluation of sample distributions, task characteristics, and dataset attributes. By validating the advantages of the inverse quadratic function in fish population prediction tasks, this study provides a valuable reference for kernel selection in RBF neural network modeling under complex environmental conditions.

Embedding the bioenergetic parameters of the large yellow croaker into the RBF neural network not only significantly improves predictive performance but also provides compelling evidence for the value of integrating mechanism-driven and data-driven modeling strategies. By incorporating bioenergetic variables related to feeding, growth, and metabolism, the model’s input space extends beyond traditional statistical factors to include mechanistic associations grounded in clear physiological and ecological principles, thereby improving interpretability and robustness in capturing variations in the target variables. Previous studies have demonstrated that regression models relying solely on environmental variables and body weight metrics often struggle to adapt to highly dynamic physiological states, resulting in limited generalization capabilities [53]. The introduction of bioenergetic or energy balance mechanisms into feeding models can substantially improve model adaptability, underscoring the importance of mechanistic factors in enhancing generalization and ecological interpretability. Furthermore, when applied to large-scale, multi-source datasets, neural networks incorporating physiological indicators consistently exhibit higher stability and predictive accuracy than traditional statistical models [54,55,56,57,58]. These findings collectively suggest that mechanistic variables play a pivotal role in improving the cross-environmental transferability of predictive models. Building upon these insights, this study explicitly simulates the energy conversion process of fish growth, integrating this mechanism with the nonlinear feature extraction capabilities of the RBF network’s hidden layer as its core methodological approach. This approach not only improves predictive accuracy in complex scenarios but also effectively mitigates the typical “black-box” limitations associated with overfitting in purely data-driven models [59,60]. The intrinsic balance among energy intake (feeding), energy utilization (metabolism/excretion), and energy accumulation (growth) provides a continuous and biologically consistent structural prior, allowing the model to maintain high stability and predictive accuracy even in the presence of outliers or noise [53]. Consequently, integrating mechanistic input features significantly enhances the predictive performance of RBF models and provides practical insights into the fusion of ecological process modeling and machine learning methods [61,62,63].

It should be noted that periodic fish removal and handling may introduce temporary disturbances to the cage population. However, only a small proportion of fish were removed relative to the total population during each sampling event. Moreover, large yellow croaker is a schooling species exhibiting coordinated feeding behavior, and no apparent systematic change in the mean feeding rate (normalized by population size) was observed throughout the experimental period. Therefore, although temporary disturbances cannot be completely excluded, the feeding signal used for model development is considered to primarily reflect stable population-level feeding dynamics under managed aquaculture conditions [64,65,66,67,68,69]. Nevertheless, further validation under long-term commercial farming conditions with minimal operational disturbance would improve the general applicability of the proposed model.

This study also has several limitations that should be acknowledged. First, the experiment was conducted using large yellow croaker fed with floating pellets, which allowed feed intake to be quantified relatively accurately. For species fed with sinking pellets, accurate measurement of feed consumption remains more challenging, and the applicability of the proposed framework therefore requires further validation. Second, the present trial lasted for 10 weeks and was conducted using a limited number of cages and one target species, which may not fully represent the variability of long-term commercial aquaculture systems. Third, although the model incorporated multiple biological and environmental variables, the dataset did not cover all possible production conditions, such as extreme weather events, disease outbreaks, different stocking densities, and diverse feeding strategies. Therefore, the current model should be considered applicable primarily to farming conditions comparable to those investigated in the present study. Future studies should validate the model across multiple species, feed types, cage systems, production seasons, and broader hydrodynamic and environmental conditions. In addition, repeated experiments using different random seeds, cross-validation strategies, and statistical comparisons of optimization algorithms should be conducted to further assess model robustness. Integration with optical, acoustic, or behavioral sensing may also improve the generalizability and practical applicability of the proposed framework.

## 5. Conclusions

In conclusion, this study developed a bioenergetics-informed PSO-optimized RBF model for estimating fish population size in cage aquaculture using feed intake, biological variables, and environmental parameters. The proposed BE-PSO-RBF model achieved improved predictive performance compared with the standard RBF and other optimized RBF models, with a MAPE of 4.14% across 47 independent test samples. These findings suggest that feed intake-based indirect estimation can provide a feasible complementary approach for population assessment in cage farming. Further validation under broader aquaculture conditions is still required before the model can be generalized to diverse species, feed types, and production systems.

## 6. Patents

The fish population counting method proposed in this study has been granted a Chinese national invention patent (Patent No. ZL 2024 1 0716845.4).

## Figures and Tables

**Table 1:** Stocking number, initial specifications, and feed particle size of large yellow croaker at different growth stages.

Cage Number	Growth Stage	Stocking Number (ind)	Initial Body Weight (g)	Feed Particle Size (mm)
A	Juvenile Stage	3600	38.76 ± 11.63	3
B	Grow-out Stage I	1050	72.12 ± 18.04	6
C	Grow-out Stage II	1050	122.58 ± 13.34	7
D	Grow-out Stage I	1050	79.37 ± 10.51	6

**Table 2:** Parameters of the St-RBF model.

Parameter Name	Symbol	Value
Number of input variables	—	16
Number of output variables	—	1
Activation function	$\phi \left(x\right)$	Gaussian: $\phi \left(x\right)\;=\;\exp\left(-\frac{∥x-c_{i}{∥}^{2}}{2\sigma_{i}^{2}}\right)$
Hidden layer centers (Center vectors)	$c_{j}$	35 (Number of center vectors)
Width parameter (Sigma)	$\sigma_{j}$	0.2428
Output layer weights	$w_{j}$	Max: 5742.65, Min: 179.42, Mean: 1106.12

**Table 3:** Partial prediction results of fish school quantity using the St-RBF model (test set, with overall error metrics).

No.	MV (ind)	PV (ind)	RE (%)	AE (ind)	MAE	MAPE/%	RMSE
1	971	374	61.48	597	278.38	33.93	380.06
2	432	349	19.21	83			
3	957	932	2.61	25			
4	1017	401	60.57	616			
5	267	93	65.17	174			
6	792	51	93.56	741			

MV = Measured value; PV = Predicted value.

**Table 4:** Structure and fitted coefficients of energetics equations for large yellow croaker.

Item	Equation Expression	Coefficients
Fecal energy (F)	F=α·C	α = 0.1576
Excretory energy (U)	$U=\beta \cdot \left(C-F\right)$	β = 0.1051
Metabolic energy (R)	$R=\gamma \cdot W^{\delta }\cdot e^{\theta T}$	γ = 0.01403, δ = 0.77321, θ = 0.03779
Growth energy (G)	G=C−R−F−U	——

All coefficients were derived by fitting standard energetics equations using literature parameters and field-measured feeding and body weight data of large yellow croaker.

**Table 5:** Statistical distribution of relative deviation for training and test datasets.

Dataset	Sample Size	±0.05	Proportion/%	±0.1	Proportion/%
Training	186	104	55.91	154	82.80
Test	47	38	80.85	41	87.23

**Table 6:** Distribution of absolute prediction errors within defined ranges.

Error Range	Sample Size	Proportion/%
±100	47	100.00
±50	39	82.98

## Data Availability

The data presented in this study are available on request from the corresponding author. The data are not publicly available due to the organisation’s privacy policy.
